# RFCell: A Gene Selection Approach for scRNA-seq Clustering Based on Permutation and Random Forest

**DOI:** 10.3389/fgene.2021.665843

**Published:** 2021-07-27

**Authors:** Yuan Zhao, Zhao-Yu Fang, Cui-Xiang Lin, Chao Deng, Yun-Pei Xu, Hong-Dong Li

**Affiliations:** ^1^Hunan Provincial Key Laboratory on Bioinformatics, School of Computer Science and Engineering, Central South University, Changsha, China; ^2^School of Mathematics and Statistics, Central South University, Changsha, China

**Keywords:** single-cell RNA sequencing, gene selection, permutation, random forest, clustering

## Abstract

In recent years, the application of single cell RNA-seq (scRNA-seq) has become more and more popular in fields such as biology and medical research. Analyzing scRNA-seq data can discover complex cell populations and infer single-cell trajectories in cell development. Clustering is one of the most important methods to analyze scRNA-seq data. In this paper, we focus on improving scRNA-seq clustering through gene selection, which also reduces the dimensionality of scRNA-seq data. Studies have shown that gene selection for scRNA-seq data can improve clustering accuracy. Therefore, it is important to select genes with cell type specificity. Gene selection not only helps to reduce the dimensionality of scRNA-seq data, but also can improve cell type identification in combination with clustering methods. Here, we proposed RFCell, a supervised gene selection method, which is based on permutation and random forest classification. We first use RFCell and three existing gene selection methods to select gene sets on 10 scRNA-seq data sets. Then, three classical clustering algorithms are used to cluster the cells obtained by these gene selection methods. We found that the gene selection performance of RFCell was better than other gene selection methods.

## Introduction

Single cell RNA-Seq (scRNA-Seq) provides unprecedented insight into biological concerns at the level of individual cells (Hwang et al., 2018). Bulk RNA sequencing analysis, based on the average expression of large populations of cells, is difficult to reveal the expression heterogeneity between different cells. However, scRNA-Seq only studies the expression of single-cell level, so scRNA-Seq improves cell resolution across global transcriptome profile (Pouyan and Kostka, 2018). In recent years, scRNA-seq has been widely used in many aspects of biological and medical research (Hedlund and Deng, 2018), for example, discovering the new cell states and tracing the origin of its development (Trapnell, 2015), cell type identification (Xu and Su, 2015), heterogeneity of cell responses (Pollen et al., 2014), understanding of cell-specific biological characteristics (Poirion et al., 2016), building gene regulatory networks across the entire gene expression profiles (Zheng et al., 2019), tracking of different cell lineage trajectories (Shao and Hofer, 2017), and cell fate decisions (Goolam et al., 2016). In addition, scRNA-seq data is useful to study cellular immunity, drug and antibiotic resistance (Patel et al., 2014).

Genome-wide transcriptome analysis is usually used to study the expression of tissue, disease and cell type-specific genes, but generating expression profiles at single-cell resolution is technically challenging. Therefore, researchers have proposed many sequencing technologies, such as: a robust mRNA-Seq protocol that is applicable to a single cell level; and a scalable method to characterize many cell types and states under various conditions and disturbances Drop-seq protocol for complex organizations (Ramskold et al., 2012; Macosko et al., 2015)). From the perspective of scRNA-Seq technology, the scRNA-seq capture efficiency and dropout rate have limitations due to the small amount of starting materials. At the same time, due to the uncertainty of cell separation protocol, library preparation methods, sequencing methods, reagent usage methods, and various types of samples, batch effects may be introduced, which leads to the high noise characteristics of scRNA-seq data (Chen et al., 2019). From the perspective of gene expression, gene expression in scRNA-Seq data is specific (Aevermann et al., 2018), only a small part of the genes are biologically meaningful. So, scRNA-Seq research is challenging due to its high noise, high dimensionality and sparsity (Schnable et al., 2009). Considering that scRNA-seq data play an important role in the effectiveness and accuracy of downstream analysis, the most important goal of scRNA-seq is to select highly variable genes in the single cell transcriptome profiling.

scRNA-seq data usually has the problems of high noise, high dimensionality and sparseness. Therefore, before downstream analysis, researchers usually use certain feature selection methods to extract scRNA-seq data. A common gene selection strategy for high-dimensional gene expression analysis is by projecting data points from a high-dimensional gene expression space into a low-dimensional space. Single cell expression data in low-dimensional space is expected to be an important feature in high-dimensional space. In recent years, there have been many methods to analyze and study scRNA-seq data from the angles of reduce dimension. Principal component analysis (PCA) (Lever et al., 2017) is a method of converting scRNA-seq data into fewer features to achieve data dimensionality reduction. By generating two-dimensional embedding of high-dimensional data, t-distributed stochastic neighborhood embedding (t-SNE) (Linderman and Steinerberger, 2019) is an effective non-linear dimensionality reduction technology that has attracted more and more scientific attention. Recently, it has been widely popular in the field of scRNA-seq data research.

Andrews and Hemberg (2019) proposed a gene selection method called M3Drop. Wang et al. (2019) proposed a new marker selection strategy SCMarker to accurately delineate cell types in scRNA-seq data by identifying genes that have bi/multi-modally distributed expression levels and are co-or mutually-exclusively expressed with some other genes. In addition, Expr is also a gene selection method based on scRNA-Seq sequencing data. This method only retains the genes with the highest average expression (logarithmic normalized count) value in all cells.

We propose RFCell, a gene selection strategy based on permutation and random forest, which uses supervised classification in pattern recognition to determine the best subset of genes for cell type recognition without referring to any known transcriptome profile or cell related information. The central idea of our method is that random forests based on ensemble method can not only process scRNA-seq data with high-dimensional features, but also evaluate the importance of each gene in gene expression data through information gain. Our main goal is to identify marker genes from scRNA-seq data that can not only judge cell types but also have biological significance. After using RFCell for gene selection on 10 scRNA-seq data sets, we found that the accuracy of the average results is higher than that of using conventional gene selection strategies.

## Materials and Methods

**FIGURE 1:**
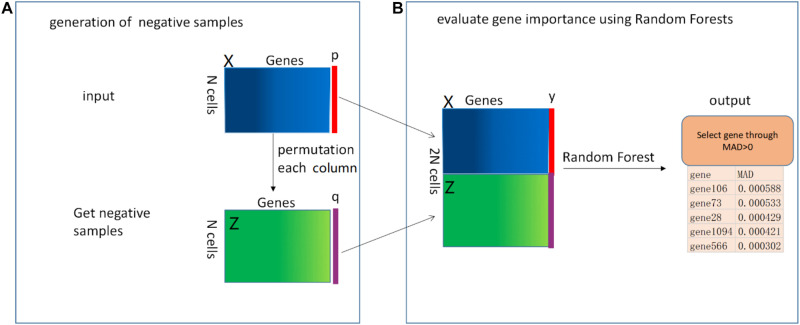
The mechanism of RFCell (scRNA-seq gene selection based on permutation and Random Forest) algorithm. The input is a gene expression matrix. The RFCell algorithm includes two steps: **(A)** Generation of negative samples; **(B)** Evaluation of gene importance using Random Forest.

The pipeline of our proposed RFCell is depicted in Figure 1. In the following section, we describe this pipeline in detail.

### Method

Pouyan and Kostka (2018) proposed RAFSIL, a random forest-based method that can learn the similarity between cells from scRNA-seq data. RAFSIL consists of two steps: feature construction based on scRNA-seq data and similarity learning. RAFSIL has strong adaptability and scalability, and the similarity can be used for typical exploratory scRNA-seq data research, such as dimensionality reduction, visualization and clustering. Considering that RAFSIL uses permutation to generate similarity, we propose to use permutation to generate negative samples. We develop RFCell, a supervised gene selection strategy based on permutation and random forest. RFCell evaluates the importance of each gene through random forest classification. RFCell works in two steps: generation of negative samples and evaluation of gene importance using Random Forest.

#### Generation of Negative Samples

It is well known that scRNA-seq data is complex and diverse, so it is particularly important for scRNA-seq data gene selection. First, to generate a random negative sample matrix of gene expression data, we input the gene expression matrix **X** (**X** consists of *m* rows and *n* columns) obtained after data preprocessing as a positive sample. After that, the gene in each column of the positive sample matrix **X** is randomly permutated to form a new gene expression matrix **Z** (**Z** consists of m rows and n columns). We define each row of cells in the new gene expression matrix **Z** as a negative sample.

Next, we create the vector **y**. First, we define the label of the positive sample matrix **X** as a vector *p*, and *p* are all 1, where the number of 1 is the number of rows (m) of the positive sample matrix **X**. Second, the label of the negative sample matrix **Z** is defined as a vector *q*, and *q* is all 0, where the number of 0 is the number of rows (m) of the negative sample matrix **Z**. Here, we convert the *p* vector and *q* vector into data frame format respectively. Third, the vector *y* (*y* consists of 2 × m rows and one column) is generated by vertically merging the vector *p* and the vector *q*.

Finally, the positive sample matrix **X** and the negative sample matrix **Z** obtained from the above are merged vertically to obtain a new gene expression matrix **N** (**N** contains 2 × m rows and *n* columns).

#### Evaluation of Gene Importance Using Random Forest

We use the randomforest (Xin-Hai, 2013) package in R language to evaluate gene importance. First, in order to generate the random forest training data set, we horizontally merge the matrix *N* and the vector *y*. Through merging, we get the random forest training data set matrix *M* (*M* contains 2 × m rows and *n*+1 columns). Then, we call the random forest R language package. According to the usage of the randomforest package in R language, we use the vector **y** obtained above as the formula setting of the randomforest package, and use the matrix *M* as data setting of randomforest package. The importance parameter is set to True, and the remaining parameters are default values.

After calling the randomforest package, we use the importance function to calculate the importance of each gene, and obtain the importance of each gene through the mean decrease accuracy (MDA). MDA represents the degree of reduction in the accuracy of random forest prediction after one gene is permutated. The larger the value, the greater the importance of the gene. In our study, genes with MDA>0 are selected as genes that can identify cell types.

### ScRNA-Seq Datasets

We tested 10 published scRNA-seq datasets and obtained results using gene selection methods. All these data sets have been used for performance research by several latest algorithms. For each data set, we use the expression unit provided by the author.

Darmanis dataset (Darmanis et al., 2015): In order to capture the cellular complexity of adult and fetal human brains at the entire transcriptome level, the authors performed single-cell RNA sequencing on 466 cells. This data set consists of oligodendrocytes, astrocytes, microglia, neuronal cells, endothelial cells, neural progenitor cells, quiescent newborn neurons, and two types of cells containing more than one different cell type Cells with characteristic genes are composed together.

Deng dataset (Deng et al., 2014): The authors used the Smart-seq or Smart-seq2 platform to perform RNA-Seq sequencing on Mus musculus cells from zygotic to late blastocysts of a single cell from the adult liver. The cells in this data set are separated from mouse embryonic oocytes to blastocyst stage, including four 1- cells (zygotes), eight early 2- cells, 12 metaphase 2- cells, 10 late 2- cells, and 14 4- cells, 28 8- cells, 50 16- cells, 43 early blast cells, 60 mid blast cells, and 30 late blast cells.

Engel dataset (Engel et al., 2016): The authors analyzed purified populations of thymic natural killer T cells (NKT cells) at the transcriptome level and epigenome level, as well as by single-cell RNA sequencing. The data consists of NKT1 cells, NKT2 cells, and NKT17 cells.

Grover dataset (Grover et al., 2016): Using single-cell RNA-seq technology, the authors systematically compared single hematopoietic stem cells (HSC) from young mice and old mice that were transgenic from Vwf-EGFP bacterial artificial chromosomes (BAC). By analyzing HSC transcriptome and HSC function at the single cell level, the authors found that molecular platelet priming and increased functional platelet bias are the main age-dependent changes in HSCs.

Pollen dataset (Pollen et al., 2014): Using microfluidic technology, the authors captured 301 single cells from 11 populations and analyzed the single-cell transcriptome within the down-sampling sequencing depth range. They proved that for unbiased cell type classification and biomarker identification, shallow scRNA-seq is indeed sufficient.

Sasagawa dataset (Sasagawa et al., 2013): The authors proposed a novel scRNA-seq method named Quartz-Seq. They applied this method to ES cells in different three cell-cycle phases (G1, S, and G2/M).

Ting dataset (Ting et al., 2014): The authors applied a microfluidic device to isolate Circulating tumor cells (CTCs) based on the model from a pancreatic cancer mouse to determine the heterogeneity of pancreatic CTCs. Then these CTCs were sequenced and compared to matched primary tumors, cell line controls.

Trapnell dataset (Trapnell et al., 2010): The author sequenced and analyzed more than 430 million paired 75 bp RNA-Seq reads from mouse myoblast cell lines on differentiation time series.

Treutlein dataset (Treutlein et al., 2014): The authors analyzed 198 single-cell transcriptomes from mouse lung epithelium in total. For time point E18.5, three individual experiments were performed using three different pregnant mices (3 biological replicates): 20 single cell transcriptomes yielded from pooled sibling lungs, 34 single cell transcriptomes yielded from one single embryonic lung, 26 single cell transcriptomes yielded from pooled sibling lungs. The authors used an unbiased genome-wide approach and classified these 80 cells into five populations: Clara (Scgblal), ciliate (Foxjl), AT1 (Pdpn, Ager), AT2 (Sftpc, Sftpb), and alveolar bipotential progenitor (BP) cells.

Zhou dataset (Zhou et al., 2016): The author used effective surface markers to capture the newborn pre-HSC with high purity, and then applied single-cell RNA sequencing to analyze endothelial cells, CD45- and CD45+ pre-HSC in the aorta-gonad-mesonephrine region, and fetus HSC of the liver.

The summary description of the scRNA-seq datasets we used is shown in Table 1.

**TABLE 1:** Summary description of the ten scRNA-seq datasets.

**Datasets**	**#Samples**	**#Genes**	**#Classes**	**Unit**
Darmanis (Darmanis et al., 2015)	466	22,088	9	CPM
Deng (Deng et al., 2014)	259	22,958	10	RPKM
Engel (Engel et al., 2016)	203	23,342	4	RPKM
Grover (Grover et al., 2016)	135	15,181	2	CPM
Pollen (Pollen et al., 2014)	249	14,805	11	TPM
Sasagawa (Sasagawa et al., 2013)	23	36,807	3	FPKM
Ting (Ting et al., 2014)	149	29,018	7	CPM
Trapnell (Trapnell et al., 2010)	372	47,192	4	FPKM
Treutlein (Treutlein et al., 2014)	80	23,271	5	FPKM
Zhou (Zhou et al., 2016)	181	23,624	8	FPKM

### Performance Evaluation

In order to compare the clustering results of RFCell and other gene selection methods, we used two commonly used clustering algorithm evaluation indicators: normalized mutual information (NMI) (Kiselev et al., 2017) and adjusted rand index (ARI) (Rand, 1971).

Mutual information (MI) measures the correlation between two sets of events. In information theory, a useful measure of information can be seen as the amount of information contained in a random variable about another random variable, or the uncertainty reduced by knowing another random variable. Formally, the MI of two discrete random variables X and Y can be defined as:

(1) $$I\left(X:Y\right)=\sum_{y\in Y}\sum_{x\in X}p\left(x,y\right)\log\left(\frac{p\,\left(x,y\right)}{p\,\left(x\right)\,p\,\left(y\right)}\right)$$

where *p*(*x*,*y*) is the joint probability distribution function of *X* and *Y*, and *p*(*x*) and *p*(*y*) are the marginal probability distribution functions of *X* and *Y*. NMI is to place MI between [0, 1] through information entropy, and its purpose is to evaluate the quality of the algorithm. For a random variable X, its information entropy can be calculated as:

(2) $$H\,\left(X\right)=\sum_{i=1}^{n}p\,\left(x_{i}\right)\,I\,\left(x_{i}\right)=\sum_{i=1}^{n}p\,\left(x_{i}\right)\,\log\frac{1}{p\,\left(x_{i}\right)}$$

The value of the random variable X = {*x*_1_,*x*_2_,…*x*_*n*_} and *p*(*x*_*i*_) represent the probability of event occurring, on the other hand, the value of random variable Y = {*y*_1_,*y*_2_,…*y*_*n*_} and *p*(*y*_*i*_) represents the probability of event occurring. NMI can be defined as:

(3) $$U\,\left(X,Y\right)=2\,\frac{I\,\left(X;Y\right)}{H\,\left(X\right)+H\,\left(Y\right)}$$

NMI is used to evaluate the consistency between the clustering results obtained and the true cell markers.

Rand Index (RI) is a measure of the similarity between clustering results and real categories. Mathematically, the RI is associated with accuracy. Given a set of S with n elements, then compare the two partitions M, N of S. The RI is calculated as follows:

(4) $$R\,I=\frac{a+b}{a+b+c+d}=\frac{a+b}{C_{n}^{2}}=\frac{a+b}{n\,\left(n-1\right)/2}$$

where a is the number of pairs of elements in S that are in the same subset in M and in the same subset in N; b is the number of pairs of elements in S that are in different subsets in M and in different subsets in N; c is the number of pairs of elements in S that are in the same subset in M and in different subsets in N; d is the number of pairs of elements in S that are in different subsets in M and in the same subset in *N*.

The RI is between [0, 1]. The greater the RI value, the more consistent the clustering result of the algorithm is with the known label, the higher the accuracy of the clustering effect, and the higher the purity in each category. The problem with the RI is that, when comparing multiple clustering results, RI values are usually high, resulting in a poor evaluation of the superiority of the clustering algorithm. Therefore, ARI presented has better differentiation degree than RI. The range of ARI is (−1, 1). ARI can be defined as:

(5) $$A\,R\,I=\frac{R\,I-E\,\left(R\,I\right)}{\max\left(R\,I\right)-E\,\left(R\,I\right)}$$

where *E*(*RI*) and *max*⁡(*RI*) can be defined as:

(6) $$E\left(RI\right)=E\left(\sum_{i,j}{(}_{2}^{n_{i,j}})\right)=\left[\sum_{i}{(}_{2}^{n_{i}})\sum_{j}{(}_{2}^{n_{j}})\right]/{(}_{2}^{n})$$

(7) $$\max\left(RI\right)=\frac{1}{2}\left[\sum_{i}{(}_{2}^{n_{i}})+\sum_{j}{(}_{2}^{n_{j}})\right]$$

where *n*_*i*,*j*_ are values from the contingency table, *n*_*i*_ is the sum of the i-th row of the contingency table, *n*_*j*_ is the sum of the j-th column of the contingency table.

Adjusted rand index is commonly used to assess the consistency between predicted clusters and true categories.

## Results

### Comparison of RFCell With Benchmark Gene Selection Methods

To show the performance of RFCell over other gene selection methods, we used three classical clustering algorithms: Clustering method for single-cell interpretation through multikernel learning (SIMLR) (Wang et al., 2017), Single-cell consensus clustering (Wilkerson and Hayes, 2010) merges clustering results of multiple cells by consensus method (SC3) (Kiselev et al., 2017) and *k*-means (Kim et al., 2019). SIMLR is a software that learns the similarity measure between cells from the input single cell data, for SIMLR, we use the SIMLR package and igraph package in R language and apply their default parameters to get a good clustering effect. SC3 is a user-friendly tool for unsupervised clustering, which methods include gene filtering, similarity calculation, Transformations, k-means, consensus clustering, and finally hierarchical clustering of the results obtained by consensus clustering. We usually use SC3, SingleCellExperiment and scater package in R language to perform SC3 clustering. For hierarchical clustering, we use the hclust (Xu et al., 2019) function with default parameters in R to perform hierarchical clustering analysis on the similarity matrix of gene expression data to obtain the final clustering results. The parameter k of three methods was set to the true number of clusters. In addition to these three algorithms, gene selection based on scRNA-seq data can apply the RFCell feature selection results to any clustering method. In fact, the final gene selected by RFCell can be used not only for any clustering algorithms, but also for similarity calculation and building a cell network. The three feature selection methods specifically for scRNA-seq data are: Andrews and Hemberg (2019) proposed M3Drop, Wang et al. (2019) proposed SCMarker. The last method of selecting genes is to select the gene with the highest average expression value (Expr). For each scRNA-seq data, we first run RFCell 10 times, and then calculate the average of the NMI and ARI as the final result.

Based on SIMLR, Table 2 clearly shows that, compared with other gene selection methods, RFCell can achieve better gene selection performance in more data in terms of NMI. For example, the average NMI of the data set clustering after RFCell gene selection is 0.593, the average NMI of the data set clustering after the Expr gene selection is 0.532, the average NMI of the data set clustering after the M3Drop gene selection is 0.544, and the average NMI of the data set clustering after SCMarker gene selection is 0.545. In more than half of all data sets, RFCell gene selection results are the best. Table 3 also shows that, compared to other feature selection methods, in terms of ARI, RFCell achieve better gene selection performance in more datasets. For example, the average ARI of the data set clustering after RFCell gene selection is 0.482, the average ARI of the data set clustering after the Expr gene selection is 0.385, the average ARI of the data set clustering after the M3Drop gene selection is 0.419, and the average ARI of the data set clustering after SCMarker gene selection is 0.398. Considering both NMI and ARI, our method does perform better than other methods on a few datasets such as the Darmanis and Engel datasets, possibly because the characteristics of the genes that can distinguish cell types for these datasets could not be captured by RFCell.

**TABLE 2:** Comparison of SIMLR performance of gene sets obtained by four gene selection methods in terms of NMI.

**DataSet**	**NMI**			
	**Expr**	**M3Drop**	**SCMarker**	**RFCell**
Darmanis	0.720	0.687	**0.727**	0.724
Deng	0.676	**0.682**	0.650	**0.682**
Engel	0.528	0.609	**0.768**	0.670
Grover	0.004	0.043	0.002	**0.084**
Pollen	0.868	**0.944**	0.908	0.938
Sasagawa	0.592	**0.621**	NA	0.595
Ting	0.781	0.706	0.767	**0.829**
Trapnell	0.102	0.127	0.066	**0.222**
Treutlein	0.425	0.411	0.433	**0.531**
Zhou	0.631	0.619	0.590	**0.663**

*NA:The number of genes selected by SCMarker is 0, so no results are obtained. The bold values mean the highest or equally-highest value among different methods.*

**TABLE 3:** Comparison of SIMLR performance of gene sets obtained by four gene selection methods in terms of ARI.

**DataSet**	**ARI**			
	**Expr**	**M3Drop**	**SCMarker**	**RFCell**
armanis	**0.549**	0.537	0.530	0.537
Deng	0.343	**0.412**	0.367	**0.412**
Engel	0.390	0.509	**0.710**	0.622
Grover	0.007	0.044	0.001	**0.109**
Pollen	0.798	**0.937**	0.832	0.917
Sasagawa	**0.561**	0.516	NA	0.555
Ting	0.540	0.532	0.491	**0.668**
Trapnell	0.010	0.062	0.010	**0.168**
Treutlein	0.237	0.239	0.285	**0.349**
Zhou	0.415	0.410	0.363	**0.483**

*NA:The number of genes selected by SCMarker is 0, so no results are obtained. The bold values mean the highest or equally-highest value among different methods.*

**FIGURE 2:**
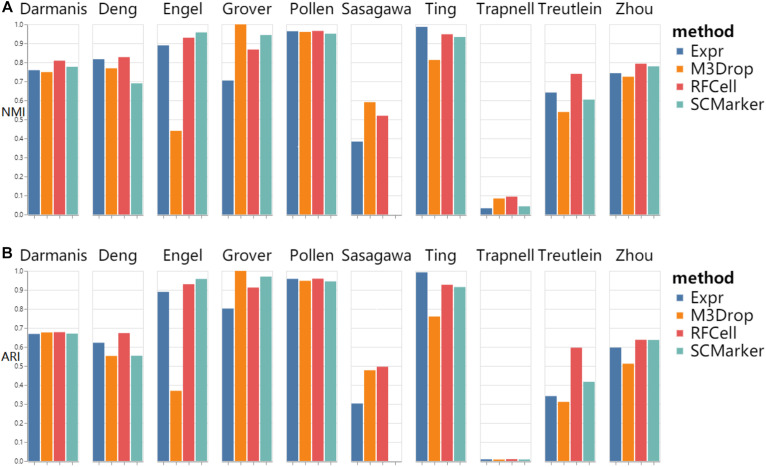
SC3 clustering results based on RFCell and three gene selection methods including Expr, M3Drop, and SCMarker in terms of NMI **(A)** and ARI **(B)**.

As shown in Figure 2, we found that RFCell basically showed good results in SC3 clustering. The picture shows that compared with other gene selection methods, the scRNA-seq data set obtained by our proposed RFCell recognizes cell types more clearly. For Darmanis dataset, Deng dataset, pollen dataset, Trapnell dataset, Treutlein dataset and Zhou dataset, compared with other gene selection methods, the gene set obtained by RFCell has obvious advantages in distinguishing cell types. Both NMI and ARI have achieved the best gene selection performance, which shows that the gene set obtained with RFCell has biological significance. For Engle dataset, Grover dataset, Sasagawa dataset and Ting dataset, we found that through different gene selection methods to obtain different gene sets have their own advantages and disadvantages in distinguishing cell types. These results indicate that scRNA-Seq data is complex and diverse, and the gene set related to cell type recognition may have some unknown factors, which require further research.

**FIGURE 3:**
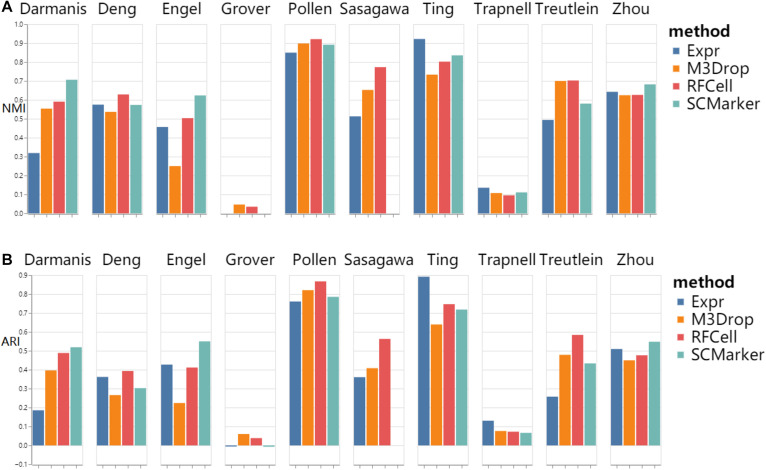
k-means clustering results based on RFCell and three gene selection methods including Expr, M3Drop, and SCMarker in terms of NMI **(A)** and ARI **(B)**.

As shown in Figure 3, we found that RFCell basically showed good results in k-means. The picture shows that compared with other gene selection methods, the scRNA-seq data set obtained by our proposed RFCell can significantly improve the clustering accuracy. For Deng dataset, pollen dataset, Sasagawa dataset and Treutlein dataset, compared with other gene selection methods, our proposed RFCell achieves satisfactory clustering performance, and more importantly, it can also provide potential biological explanations for clustering. This also shows that RFCell can identify the gene sets that contribute the most to the clusters.

### Application of RFCell to Single Cell RNA-seq Data

**FIGURE 4:**
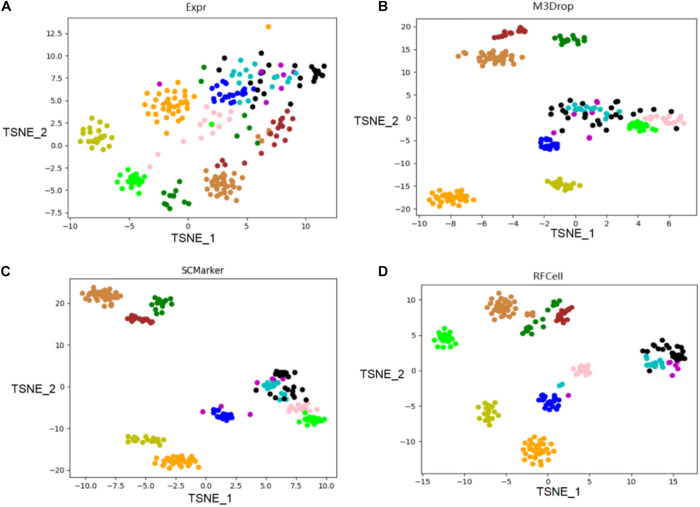
On the published pollen data of the scRNA-seq data set, the gene sets obtained by the three gene selection methods (Expr, M3Drop, and SCMarker) and the gene sets obtained by the RFCell gene selection method were compared. The visualization diagrams respectively show the gene sets obtained by the four gene selection methods: **(A)** Visualization of the results of Expr gene selection; **(B)** Visualization of the results of M3Drop gene selection; **(C)** Visualization of the results of SCMarker gene selection; **(D)** Visualization of the results of RFCell gene selection.

**FIGURE 5:**
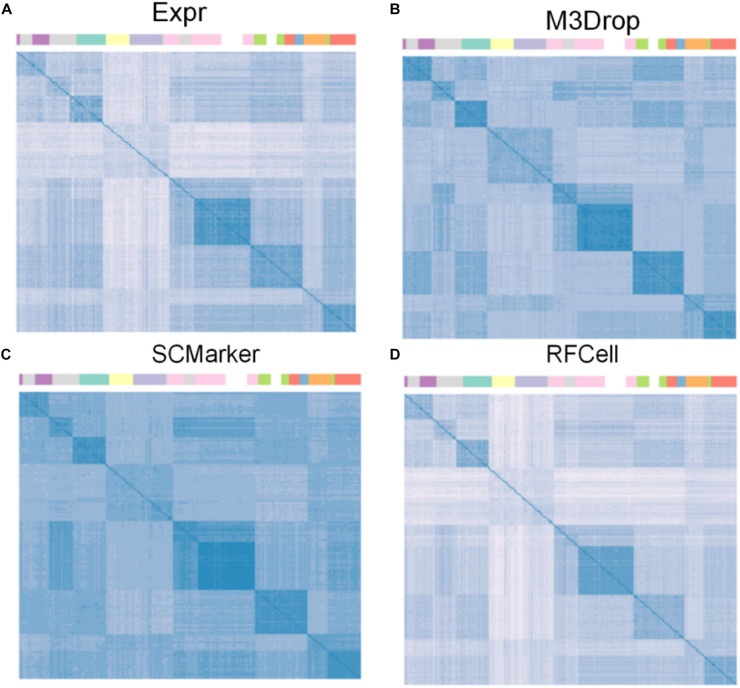
The heat map of the result is derived from the spearman similarity measure of the gene set obtained after the gene selection of pollen data by four gene selection methods. The cells in the matrix are sorted by their true labels so that cells of the same type are adjacent. Cell clusters are clearly indicated by colored bars. **(A)** Heat map of the gene set obtained by the Expr gene selection; **(B)** Heat map of the gene set obtained by the M3Drop gene selection; **(C)** Heat map of the gene set obtained by the SCMarker gene selection; **(D)** Heat map of the gene set obtained by the RFCell gene selection.

We use the single-cell transcriptome data of 249 cells captured in 11 populations obtained using microfluidic technology as our original data, and visualize the different gene sets corresponding to the original data. Data visualization results show that RFCell separates cells more clearly. It is better than the results obtained by Expr, M3Drop and SCMarker (Figures 4, 5).

As shown in Figure 4, the visualization results of the gene set selected by the Expr method show that only five cell types can be clearly distinguished, and the other cell types are scattered in confusion. The visualization results of the gene set selected by the M3Drop method also show that although there are eight cell types that can be effectively identified, the other three cell types (cell type 4, cell type 5, and cell type 6) are scattered and difficult to identify. The visualization results of the gene set selected by the SCMarker method are also difficult to effectively distinguish cell types. On the one hand, cell type 4 and cell type 5 are too widely dispersed; on the other hand, there is multiple cell types (cell type 3, cell type 4, cell type 5, and cell type 6) has a crossover, which makes the identification of cell type confused. The result of the visualization of the gene set obtained after gene selection by our proposed RFCell shows that all cell types can be clearly identified, and there is no crossover between cell types. This also shows that RFCell has superiority in cell type recognition. The heat map in Figure 5 is derived from the spearman similarity measure of the gene set obtained after gene selection of pollen data by four gene selection methods. RFCell also showed better performance.

## Discussion and Conclusion

In recent years, scRNA-seq technology has become a powerful tool for studying cell heterogeneity in tissues, advances in sequencing technology have enabled scientists to perform large-scale transcriptome profiling at single cell resolution in a high-throughput manner, clustering algorithms have passed unsupervised learning has become the main way to identify and characterize new cell types and gene expression patterns, however, on the one hand, differences in scRNA-seq technology can cause noise in scRNA-seq data, especially because it is impossible to repeat measurements on the same cell (Severson et al., 2018; Zhang et al., 2020). On the other hand, scRNA-seq data is noisier and more complex than traditional RNA-Seq data, and the high variability of the data also brings challenges to scRNA-seq data analysis (Chen et al., 2019). In order to analyze scRNA-seq data, feature selection methods can greatly reduce the dimensionality of the data and improve the results of cell type recognition. For analyzing specific data, especially gene expression data, many studies have shown that certain gene sets with correlation and functional synergy play an important role in analyzing scRNA-seq data and identifying specific cell types (Eisen, 1998; Young et al., 2010; Buettner et al., 2017).

In this study, we proposed a new feature selection method, RFCell, for gene selection of scRNA-seq data. Through feature selection based on permutation and random forest for each gene expression data. RFCell uses classic machine learning methods to perform supervised classification of scRNA-seq data to show its superiority compared with other feature selection methods. RFCell is characterized by a series of noteworthy functions. First, the negative samples are obtained by using scRNA-seq data permutation. Secondly, RFCell obtains the training data of the random forest by combining the original scRNA-seq and negative samples. Third, considering that the information contained in each genome and the ability to recognize cell types is different, we estimate the importance of each genome by calculating the importance function. Finally, RFCell selects genes with MDA>0 as the gene set that can identify cell types. This is done to make the results of RFCell robust to gene set mutations.

RFCell does have some limitations. First of all, the negative samples obtained from the original gene expression data using permutation are uncertain, so this means that for each data set, there may be some genes that can identify cell types are disrupted to the wrong cells. Therefore, in this process, some genes that are essential for classification are likely to be discarded, resulting in failure to obtain the best classification results. With this in mind, we have conducted many experiments to make RFCell stable to the results of gene selection. Experiments include visual analysis of gene sets obtained through different gene selection methods. The details are as follows. We use the single-cell transcriptome data of 249 cells captured in 11 populations obtained using microfluidic technology as our original data, use four gene selection methods to select the gene sets of the original data to obtain different gene sets, and visualize these sets of genes. In addition, we also do heat map analysis on gene sets. Corresponding experimental results show that RFCell shows superiority in the visualization map, but RFCell needs to be improved in the heat map analysis.

It is expected that biological information (such as labeled gene sets) will be used in the future to select genes related to cell types in scRNA-seq for further study. Incorporating information from different views may be helpful in improving gene selection (Liu et al., 2020a; Liu et al., 2020b; Lan et al., 2020). There are some differences among the results for scRNA-seq data based on different gene selection methods. Analyzing the preference performance of different gene selection methods for scRNA-seq data could improve the accuracy of cell type identification. Therefore, we believe that integrating different gene selection methods may benefit gene selection.

## Data Availability Statement

Publicly available datasets were analyzed in this study and the references for the data are provided in this article.

## Author Contributions

H-DL conceived the study. YZ and Z-YF performed the experiments and wrote manuscripts. C-XL, CD, Y-PX, and H-DL wrote the manuscript. All authors contributed to the article and approved the submitted version.

## Conflict of Interest

The authors declare that the research was conducted in the absence of any commercial or financial relationships that could be construed as a potential conflict of interest.

## Publisher’s Note

All claims expressed in this article are solely those of the authors and do not necessarily represent those of their affiliated organizations, or those of the publisher, the editors and the reviewers. Any product that may be evaluated in this article, or claim that may be made by its manufacturer, is not guaranteed or endorsed by the publisher.
